# The effect of berberine chloride and/or its combination with vancomycin on the growth, biofilm formation, and motility of *Clostridioides difficile*

**DOI:** 10.1007/s10096-020-03857-0

**Published:** 2020-03-05

**Authors:** Dorota Wultańska, Michał Piotrowski, Hanna Pituch

**Affiliations:** Department of Medical Microbiology, Medical University of Warsaw, Warsaw, Poland

**Keywords:** *Clostridioides difficile*, Biofilm formation, Berberine chloride (BBR), Vancomycin (VA), Synergistic effect, Inhibition of motility

## Abstract

This study aims to investigate the antimicrobial and antibiofilm activity of berberine chloride (BBR) and vancomycin (VAN) as well as synergistic combinations of BBR with VAN against *Clostridioides difficile* strains. The effect of different concentrations of BBR on strain motility was also assessed. Twelve *C. difficile* strains (two reference *C. difficile* 630, ATCC 9689, and one control M120, and 9 clinical *C. difficile* strains belonging to the PCR-ribotype (RT027)) were collected and investigated for their susceptibility to BBR and VAN in planktonic and biofilm forms. Both the minimum inhibitory concentration (MIC) and the minimum bactericidal concentration (MBC) of BBR for the *C. difficile* strains were found to vary over a broad range (256–1.024 mg/L and 256–16.384 mg/L, respectively). The MIC and MBC of VAN also varied greatly, ranging from 0.25 to 4.0 mg/L for MIC and 0.25 to 64.0 mg/L for MBC. The synergistic effect of the sub-MIC (1/2 MIC) BBR with VAN reduced of MICs of VAN against the planktonic forms of ten *C. difficile* strains. The sub-MIC of BBR enhanced the biofilm formation of one strain and was found to be statistically significant. In addition, the sub-MIC of BBR with VAN surprisingly enhanced the biofilm formation of one *C. difficile* strain. The effect of inhibition of motility in the presence of BBR was statistically significant for 3 clinical strains (*p* < 0.05). Altogether, BBR exhibited strong antimicrobial activity against *C. difficile*, and the analysis of the combination of BBR with VAN showed a synergistic effect.

## Introduction

*Clostridioides difficile* (formerly *Clostridium difficile*) is one of the most common multi-drug-resistant organisms in hospital-acquired infections and is associated with high morbidity and mortality [1–3]. The main virulent factors of *C. difficile* are two toxins: toxin A (TcdA) and toxin B (TcdB). These virulence factors are glycosyltransferases that inactivate Rho, Ras, and Rac (GTP-binding proteins), resulting in the damage of the colonic epithelium and, subsequently, diarrhea [4]. Some strains possess a third toxin, called binary toxin (CDT) [1]. The increase of the incidence of *Clostridioides difficile* infections (CDI) is predominantly due to hyperepidemic strains of the genotype NAP1/BI/RT027/toxinotype III, which emerged at the beginning of the new millennium [5]. In a survey of CDI organized in Polish hospitals (2011–2013), *C. difficile* strains belonging to RT027 were the most prevalent PCR-ribotype in all hospitals involved in the study [6]. Strains belonging to RT027 are characterized by a higher production of toxin A (TcdA) and toxin B (TcdB) in vitro and the presence of binary toxin genes (*cdtA* and *cdtB*) [1, 4]. These proteins are the main virulent factors in pathogenesis of CDI. Other factors, such as fibronectin-binding protein A, surface layer proteins (SPLs), cell-wall proteins (CWPs), flagella, and cysteine protease Cwp84, have also been shown to be involved in the adhesion and colonization of the gut by *C. difficile*. Among these, Cwp84, flagella, LuxS protein, and surface layer proteins (SLPs) are especially associated with biofilm formation. A microbial biofilm is defined as a structured consortium of microbial cells surrounded by the self-produced matrix. Biofilms contribute to the tolerance of *C. difficile* to antibiotics, including those used as the first and second lines of the treatment for patients with CDI [7]. Metronidazole and vancomycin are the drugs of choice for the treatment of CDI; however, they are associated with a high incidence of relapse [8]. Although most *C. difficile* isolates are still susceptible to metronidazole and vancomycin, the resistance and reduced sensitivity of *C. difficile* to these drugs have been recently reported [9, 10]. Recurrent or persistent CDI have also been linked to the ability to produce biofilms [11]. Given the poor efficacy of standard treatment for frequent recurrences of CDIs, researchers have been actively searching treatment alternatives for several decades.

Berberine is a polyphenolic compound and a plant alkaloid that is isolated from *Coptis chinensis* (Chinese goldthread), *Hydrastis canadensis* (goldenseal), *Rhizoma coptidis*, Cortex phellode, and berberis [12]. Berberine chloride (BBR) is a quaternary ammonium isoquinoline alkaloid and is the most commonly available salt form of berberine [13]. Berberine is strong yellow in color and emits a strong yellow fluorescence under ultraviolet light [13]. BBR exhibits many biological functions and has been used to treat gastroenteritis, bacterium-associated diarrhea, and intestinal parasitic infections [14, 15]. The effect of berberine on *C. difficile* and in the treatment of CDI has been previously discussed in the literature [16–18]. Berberine in combination with ciprofloxacin was found to reduce the formation of biofilms by multi-resistant *Salmonella* sp. strains [19]. In this study, we investigated the antimicrobial and antibiofilm activity of synergistic combinations of berberine (BBR) and vancomycin (VAN) against *C. difficile* strains and the activity of BBR and VAN individually, as well as the effect of different concentrations of BBR on strain motility.

## Materials and methods

### Bacterial strains

Twelve *C. difficile* strains were used in this study: two reference strains (*C. difficile* 630 (RT012) and ATCC 9689 (RT001)), one control strain (*C. difficile* M120 (RT078)), and nine clinical strains consisting of clinical isolates and toxigenic strains belonging to the PCR-ribotype (RT027). The original numbers of all the clinical *C. difficile* strains were anonymized and designated a number from 4 to 12. The clinical *C. difficile* strains were previously isolated from patients with diarrhea admitted to Polish hospitals between 2012 and 2013. All *C. difficile* strains were collected in the Anaerobic Laboratory, in the Department of Medical Microbiology, at the Medical University of Warsaw. *C. difficile* strains were stored at − 70 °C in a Microbank™ bacterial storage system (Pro-Lab Diagnostics, UK) until use in the experiment. *C. difficile* 630 and M120 were kindly provided as a gift from Prof. Brendan Wren, in the Department of Pathogen Molecular Biology, at the London School of Hygiene and Tropical Medicine, London, UK. Strain ATCC9689 was purchased from bioMérieux (Marcy l’Etoile, France). The strains were thawed before use in experiments, cultured on Columbia blood agar solid medium (bioMérieux, Marcy l’Etoile, France) at 37 °C for 24 h under anaerobic conditions. All isolates were cultivated in brain–heart infusion (BHI; Difco, USA) medium at 37 °C under anaerobic conditions, unless stated otherwise.

### Chemicals

Berberine chloride (BBR) (chemical formula C20H18CINO4) purchased from Sigma-Aldrich Co., Ltd. (St. Louis, MO, USA) was dissolved in DMSO (Biomus, Poland) and filtered through a 0.22-μm Millipore filter (Corning, USA). Vancomycin (VAN) was obtained from Sigma-Aldrich Co., Ltd. (St. Louis, MO, USA). BHI medium was obtained from (BIOMAXIMA, Lublin, Poland).

### Determination of the minimal inhibitory concentration and minimal bactericidal concentration of berberine chloride and vancomycin for *C. difficile* strains

The minimal inhibitory concentrations (MICs) of berberine hydrochloride (BBR) and vancomycin (VAN) for the strains were determined using the broth microdilution method in a 96-well plate (Nunc, Denmark). An initial stock solution was prepared by dissolving BBR and VAN in dimethyl sulfoxide (DMSO, Biomus, Poland). Dilutions of 16.384, 8.192, 4.096, 2.048, 1.024, 900, 640, 512, 256, 128, 64, and 32 mg/L of BBR were prepared in BHI medium (Difco, USA). Vancomycin was used at the following dilutions: 64, 32, 16, 8, 6, 4, 2, 1, 0.5, 0.25, 0.125, and 0.0625 mg/L. Wells containing 180 μL of dilution were inoculated with 20 μL of suspension 3 McFarland turbidity strains *C. difficile* and incubated at 37 °C for 48 h under anaerobic conditions. The positive control (P) was BHI medium with 20 μL of suspension 3 McFarland strains *C. difficile*, and the negative control (N) was BHI medium. All strains were tested in triplicate. Following incubation, optical density at 600 nm was measured using a microplate reader (Bio-Rad, USA). The minimal bactericidal concentration (MBC) was determined by plating the cell suspensions in 96-well plates used for MIC tests onto Columbia agar containing 5% sheep blood (Beckton Dickinson, Heidelberg, Germany) and incubated at 37 °C for 48 h under anaerobic conditions. The bacterial growth was then visually observed.

### Effect of berberine on MIC of vancomycin in *C. difficile* planktonic growth

The experiments were planned to stimulate situations in which bacteria are exposed to 1/2 MIC values (sub-MICs) of BBR with VAN. After the determination of the MICs of BBR, sub-MIC was added to the BHI medium with different concentrations of VAN. The synergism of BBR with VAN for the twelve strains was determined by the broth microdilution method in a 96-well plate. The following sub-inhibitory concentrations of BBR were prepared for individual strains: strains 2, 3, 5, 6, 7, 9, and 10: 128 mg/L of BBR; strains 11 and 12: 450 mg/L of BBR; strains 4 and 8: 320 mg/L of BBR; and strain 1: 512 mg/L of BBR, with the following concentrations of vancomycin: 64, 32, 16, 8, 4, 2, 1, 0.5, 0.25, 0.125, and 0.0625 mg/L. The solutions thus prepared were added at 180 μL to wells from 20 μL of a *C. difficile* strain suspension with a turbidity of 3 McFarland and incubated at 37 °C for 48 h. The positive control (P) was BHI medium with 20 μL suspension of 3 McFarland turbidity strains *C. difficile*, and the negative control (N) was BHI medium. All strains were tested in triplicate. After incubation, the growth of the bacteria was visualized to determine the synergistic MIC.

### Effect of sub-inhibitory concentration of berberine and vancomycin on biofilm formation

Biofilm formation was tested according to the methods described previously [20]. All *C. difficile* strains were incubated overnight in a BHI medium (Difco, USA) at 37 °C. One hundred and eighty microliters of the BHI broth was pipetted into each well of a 96-well flat-bottomed microplate (Nunc, Denmark). Subsequently, 20 μL of *C. difficile* was added (three wells for each strain). Wells with BHI broth without the inoculum were used as the controls. The following sub-MICs of BBR were prepared for individual strains 2, 3, 5, 6, 7, 9, and 10: 128 mg/L; for strains 11 and 12: 450 mg/L; for strains 4 and 8: 320 mg/L; and for strain 1: 512 mg/L. Similarly, studies on biofilm production under the influence of vancomycin were carried out. Vancomycin sub-MICs were used for individual strains: 0.125 mg/L for strains 6 and 9; 0.25 mg/L for strains 3, 5, 7, 8, 10, and 11; 0.5 mg/L for strain 12; 1.0 mg/L for strain 2; and 2.0 mg/L for strains 1 and 4.

Plates were incubated at 37 °C for 48 h under anaerobic conditions for biofilm formation. After 48 h, the liquid phase was aspired using a sterile pipette, washed twice with phosphate buffer saline (PBS) (Biomed, Poland), and air-dried at 37 °C for 15 min. Each well was then stained with crystal violet (CV) (Analab, Poland) for 10 min. The CV was removed and the wells were washed eight times with PBS. After air-drying for 15 min at 37 °C, the CV within the biofilms was dissolved in ethanol and the absorbance was measured at 620 nm (*A*_620_) using a Bio-Rad 550 Microplate Reader (Bio-Rad, USA). All strains were tested six times. The average values for each *C. difficile* strain were calculated. The effect of berberine and vancomycin on *C. difficile* biofilms was determined using CV staining of cells in the biofilms in microtiter plates, based on the study by Stepanovic et al. [21], with modifications, as described in the previous section.

### Determination of synergism for berberine with vancomycin in biofilm formation

In order to determine the formation of biofilm under the influence of sub-inhibitory doses of BBR with VAN, the following combinations of medium and agent concentrations were used: for strain 2: 128 mg/L of BBR and 1.0 mg/L of VAN; for strains 3, 5, 7, and 10: 128 mg/L of BBR and 0.25 mg/L of VAN; for strain 12: 450 mg/L of BBR and 0.5 mg/L of VAN; for strain 4: 320 mg/L of BBR and 2.0 mg/L of VAN; for strain 6 and 9: 128 mg/L of BBR and 0.125 mg/L of VAN; for strain 8: 320 mg/L of BBR and 0.25 mg/L of VAN; for strain 11: 450 mg/L of BBR and 0.25 mg/L of VAN; and for strain 1: 512 mg/L of BBR and 2.0 mg/L of VAN. All *C. difficile* strains were incubated overnight in a BHI medium at 37 °C. One hundred and eighty microliters of the BHI broth was pipetted into each well of a 96-well flat-bottomed microplate (Nunc, Denmark). Subsequently, 20 μL of *C. difficile* (three wells for each strain) was added to the wells. Wells with BHI broth without the inoculum were used as the controls. The plates were incubated at 37 °C for 48 h under anaerobic conditions for biofilm formation. After 48 h, the liquid phase was aspired using a sterile pipette, washed twice with phosphate buffer saline (PBS) (Biomed, Poland), and air-dried at 37 °C for 15 min. Each well was then stained with crystal violet (CV) (Analab, Poland) for 10 min. The CV was removed and the wells were washed eight times with PBS. After air-drying for 15 min at 37 °C, the CV within the biofilms was dissolved in ethanol and the absorbance was measured at 620 nm (*A*_620_) using a Bio-Rad 550 Microplate Reader. All strains were tested six times. The average values for each *C. difficile* strains were calculated.

### Confocal laser scanning microscopy

Biofilms were visualized via confocal laser scanning microscopy (CLSM) according to methods previously described by Piotrowski et al. [22]. Only two strains, *C. difficile* 630 and clinical strain 4, were selected for the visualization of biofilm under CLSM, as they showed the highest vancomycin minimal inhibitory concentration (MIC = 4.0 mg/L) among the strains tested. Biofilms were grown on sterile 10-mm-diameter glass-bottom dishes (Nunc, Denmark). Overnight cultures of *C. difficile* were diluted in fresh BHI with or without the sub-inhibitory concentration of VAN, BBR, and VAN with BBR. The biofilms were grown on sterile glass-bottomed flasks. As a result, 2500 μL of BHI medium and 500 μL of overnight cultures of the two strains were obtained.

The biofilms were grown by culturing the strains as follows: BHI medium supplemented with 2 mg/L of VAN for strains 630 and 4; BHI medium supplemented with 512 mg/L of BBR for strain 630; and BHI medium supplemented with 320 mg/L of BBR for strain 4. The biofilms were allowed to grow for 48 h at 37 °C under anaerobic conditions. The mature biofilms were then washed twice using 10 mM MgSO_4_ before staining with acridine orange (10 μ/mL) for 30 min in the dark. The dishes were washed twice with 10 mM MgSO_4_. Imaging was performed using a Nikon A1R MP microscope with a Nikon Ti Eclipse series (Nikon, Japan) using a × 60 objective lens and immersion oil. Images were acquired at 2040 × 2048 pixels using a Z-step of 0.1 μm. Acridine orange was detected using an excitation wavelength of 488 nm and emission wavelength of 500–550 nm. Images were processed and analyzed using NIS-Elements AR v. 4.10 software.

### Effect of a sub-inhibitory concentration of berberine on the motility of *C. difficile* in vitro

The effects of BBR on the motility of *C. difficile* were investigated in 12 strains. Among them, 3 strains were used as a control: motile 630, non-motile M120, and motility ATCC 9689 [23]. The 9 other strains consisted of clinical isolates and toxigenic strains belonging to the PCR-ribotype (RT027). After thawing, all strains were seeded on Columbia agar medium (bioMérieux, Marcy l’Etoile, France) and incubated for 48 h in an anaerobic atmosphere. For each strain, a single colony calibrated with 1 μL of *C. difficile* culture was plated onto pre-reduced 0.4% agar plates with BHI medium without BBR (as a control of motility) and with different concentrations of BBR according to the 1/2 MIC value of all the individual strains (512, 450, 320, or 128 mg/L), followed by incubating at 37 °C under anaerobic conditions. Readings were carried out after 24 h and 48 h. The inhibition of motility was assessed by measuring the diameter of the spot on the soft agar and comparing the results with the control plates (without BBR). The readings were performed independently by two separate individuals.

### Statistical analysis

At least three independent replicates of each 96-well-plate experiment were performed. Statistical analysis was performed using Statistica software (version 13, StatSoft, Poland). The normal distribution of the values was confirmed using the Shapiro–Wilk test. The effect of a sub-inhibitory concentration of BBR on the inhibition of the motility of the *C. difficile* strains was assessed using the Mann–Whitney *U* test. Differences in biofilm formation were calculated via Kruskal–Wallis one-way analysis of variance (ANOVA). Differences were considered as statistically significant for *p* values < 0.05.

## Results

### MIC and MBC values of *C. difficile* with berberine and vancomycin

The summary of results is in Table 1. Susceptibility testing showed that the MIC values of BBR ranged from 256 to 1024 mg/L (median ca. 491.3 mg/L). Likewise, the MBC values of BBR ranged from 256 to 16.384 mg/L (median ca. 1.879 mg/L). Susceptibility testing showed that the MIC values of VAN ranged from 0.25 to 4.0 mg/L (median ca. 1.2 mg/L). Similarly, the MBC values of VAN ranged from 0.25 to 64.0 mg/L (median ca. 7.3 mg/L).

**Table 1:** Minimal inhibitory concentrations and bactericidal concentrations of berberine and vancomycin and synergistic effect of berberine with vancomycin on *C. difficile* strains

No. of strain		MIC (mg/L) of BBR**	MBC (mg/L) of BBR	MIC (mg/L) of VAN**	MBC (mg/L) of VAN	Synergistic effect of sub-MICs of BBR with VAN
1	630	1.024	16.384	4.0	64.0	0.5
2	ATCC 9689	256	256	2.0	2.0	0.0625
3	M 120	256	256	0.5	1.0	0.5
4*	1468	640	640	4.0	6.0	1.0
5*	3972	256	256	0.5	0.5	0.25
6*	898	256	1024	0.25	4.0	0.0625
7*	1201	256	256	0.5	0.5	0.125
8*	5473	640	640	0.5	0.5	0.0625
9*	325	256	256	0.25	0.25	0.125
10*	443	256	256	0.5	0.5	0.125
11*	2292	900	1024	0.5	4.0	0.5
12*	5323	900	900	1.0	4.0	0.0625

*BBR* berberine, *VAN* vancomycin

*Clinical *C. difficile* strains

### Berberine decreased the MIC of vancomycin in the plankton forms of *C. difficile* growth

Our results showed that the sub-MIC values of BBR significantly reduced the MIC values of VAN for ten strains. In the case of the VAN-resistant reference strain, strain 630 (MIC = 4 mg/L) and clinical strain 4 (MIC = 4 mg/L), the MICs of VAN under the influence of BBR decreased significantly from 4.0 to 0.5 mg/L and 1.0 mg/L, respectively. The MIC value of VAN of the reference strain ATCC 9689 was found to decrease from an MIC value of 2.0 mg/L to 0.0625 mg/L. For the M120 control strain, and for one clinical strain (no. 11), the MICs of VAN did not decrease and the value remained the same, i.e., 0.5 mg/L. All results of the synergism of BBR with VAN are presented in Table 1.

### Effect of the sub-inhibitory concentration of berberine and vancomycin and berberine with vancomycin on biofilm formation

The treatment of 12 *C. difficile* strains with a sub-inhibitory concentration of BBR or VAN did not cause any significant differences in biofilm formation in strains, as follows: strain 630: BBR (*p* = 1.0) and VAN (*p* = 0.09); strain ATCC9689: BBR (*p* = 0.39) and VAN (*p* = 0.22); strain M120: BBR (*p* = 1.0) and VAN (*p* = 0.64); strain 4: BBR (*p* = 0.37) and VAN (*p* = 0.86); strain 6: BBR (*p* = 0.27) and VAN (*p* = 1.0.); strain 7: BBR (*p* = 1.0) and VAN (0.54); strain 8: BBR (*p* = 1.0) and VAN (*p* = 1. 0); strain 9: BBR (*p* = 1.0) and VAN (*p* = 1.0); strain 10: BBR (*p* = 0.22) and VAN (*p* = 1. 0); strain 11: BBR (*p* = 0.11) and VAN (*p* = 1.0); and strain 12: BBR (*p* = 1.0) and VAN (*p* = 0.92). For strain 5, a statistically significant *p* value was observed for BBR (*p* = 0.02), but not VAN (*p* = 0.99).

**Fig. 1:**
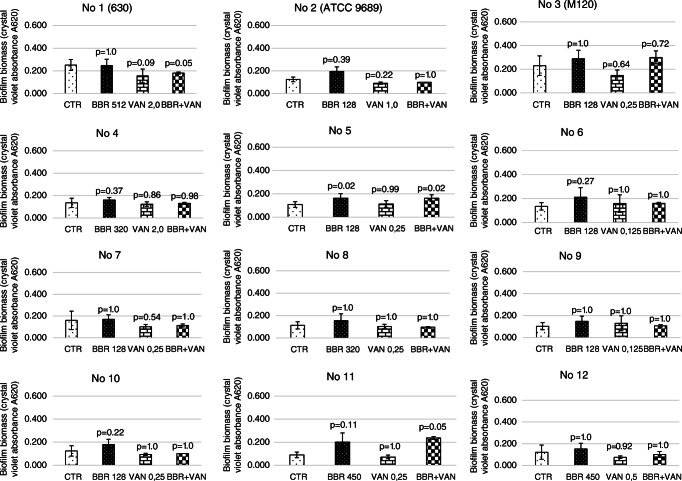
Average biofilm formation by examined *C. difficile* strains with different concentrations of BBR, VAN, and the synergism of VAN and BBR. Legend: CTR, control; BBR, berberine; VAN, vancomycin; COM, combination of berberine and vancomycin

However, statistically significant differences in biofilm formation were observed for strain no. 5 (*p* = 0.02) treated with a sub-inhibitory concentration of BBR with VAN. In addition, treatment with sub-MICs of BBR and VAN was found to induce biofilm formation of strains no. 1 (630) and no. 11 but it was not statistically significant (*p* = 0.05) (Fig. 1).

### Confocal laser scanning microscopy

**Fig. 2:**
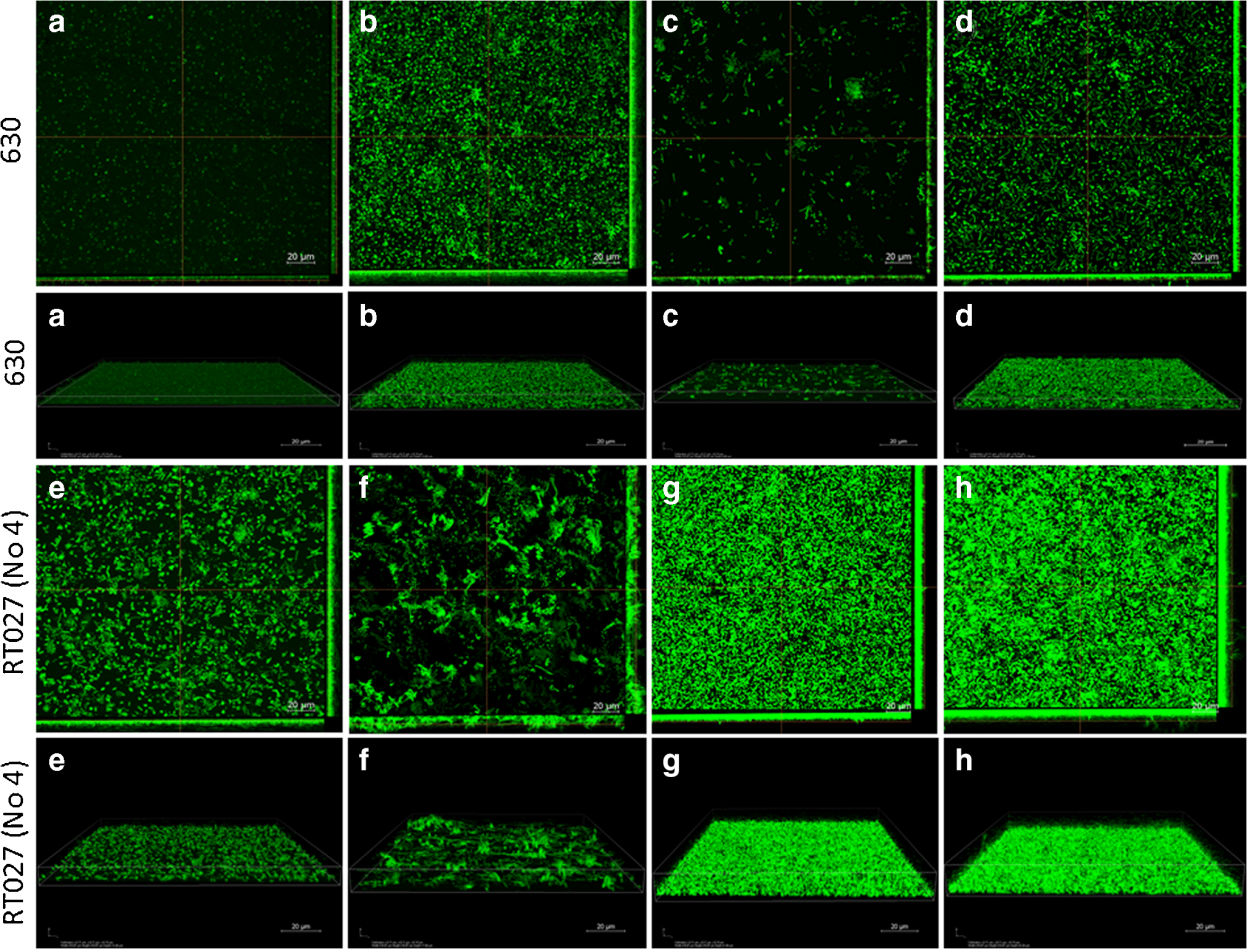
Effects of VAN and BBR and VAN with BBR on reference *C. difficile* strain 630 and clinical RT027 (no. 4) on biofilm formation. Representative confocal microscopy images of horizontal (xy) and vertical (xz) projections of *C. difficile* biofilm structures. Slices viewed with maximum intensity projection. Legend: A, control; B, VAN 2 mg/L; C, BBR 512 mg/L; D, VAN 2 mg/L with BBR 512 mg/L; E, control; F, van 2 mg/L; G, BBR 320 mg/L; *H*, VAN 2 mg/L with BBR 320 mg/l

After investigating the influence of VAN and BBR on all twelve *C. difficile* strains, two strains were selected for biofilm testing by confocal microscopy. The two strains selected had the highest MIC values of VAN among the strains. For the biofilm testing of strain 630, a sub-inhibitory dose of VAN (2 mg/L) and BBR (512 mg/L) was used. This strain in the control produced a biofilm with a very homogeneous, carpet-like, dense structure (Fig. 2A). The generation of biofilm by strain 630 in the presence of 2 mg/L VAN was also very dense, but irregular with a high 3D architecture containing microaggregates (Fig. 2B). In the presence of a sub-inhibitory dose of BBR, the biofilm produced by strain 630 was very different to that produce by the control and biofilm with VAN. In this case, the biofilm was very thin and irregular and also contained microaggregates (Fig. 2C). The biofilm of strain 630 in 2 mg/L of VAN and 512 mg/L of BBR was thick, heterogeneous, and irregular with a small amount of microaggregates (Fig. 2D). Sub-inhibitory doses of VAN (2 mg/L) and BBR (320 mg/L) were also used to test a second strain of *C. difficile*: strain 4. This strain produced an irregular, heterogeneous biofilm control with a fairly high 3D architecture containing microaggregates (Fig. 2E). Under the influence of a sub-inhibitory dose of VAN, this strain produced a very thick, but rare, irregular biofilm with a 3D high architecture containing microaggregates (Fig. 2F). In the presence of BBR, this strain produced a biofilm with a regular structure and fairly high 3D architecture (Fig. 2G). The biofilm of strain 4 with 2 mg/L of VAN and 320 mg/L BBR was rather homogeneous and thick with a very high 3D architecture containing microaggregates (Fig. 2H).

### Sub-inhibitory concentrations of berberine inhibit motility of *C. difficile* strains

**Fig. 3:**
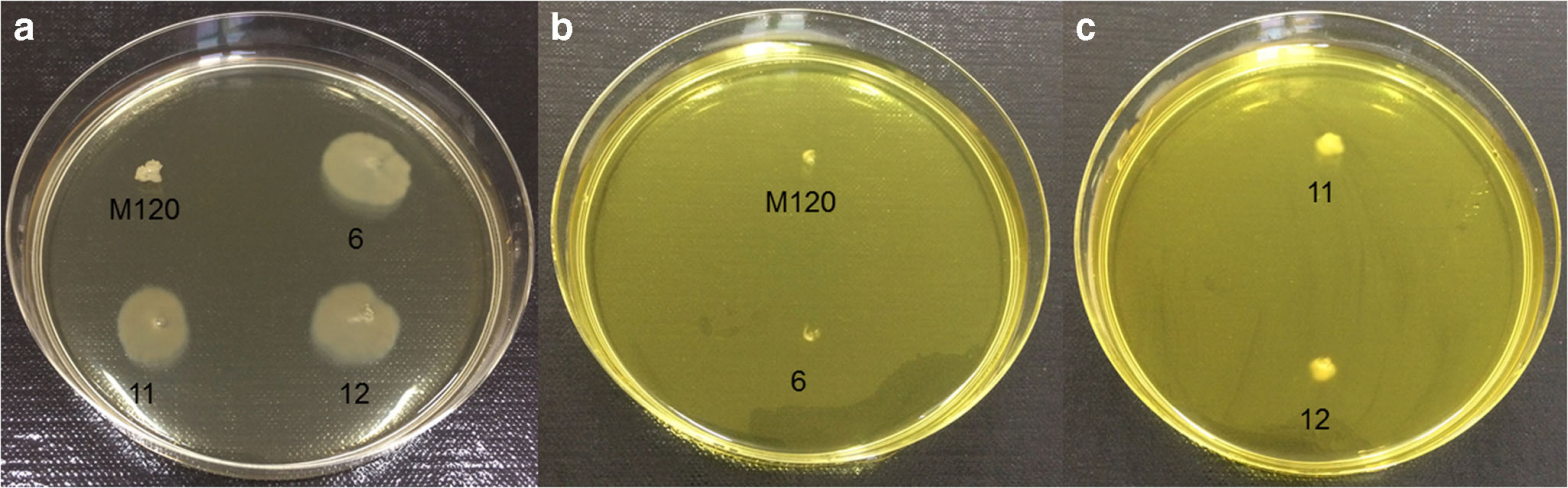
Effect of a sub-inhibitory concentration of berberine on the motility of *C. difficile* in vitro. Legend: A, control (without BBR); B and C, with sub-MICs of BBR: 128 mg/L or 450 mg/L, respectively

Three clinical *C. difficile* isolates (4, 11, and 12) showed inhibition of motility after the addition of sub-inhibitory (1/2 MIC) doses of BBR (320, 450, and 450 mg/L, respectively) to the BHI agar plates. This effect was statistically significant for strain 4 after 48 h (*p* < 0.01), for strain 11 (24 h, *p* = 0.016; 48 h, *p* = 0.02), and strain 12 (24 h, *p* = 0.01; 48 h, *p* < 0.01) (Table 2). The addition of BBR at 1/2 MIC on strains 630 (512 mg/L) and ATCC 9689 (128 mg/L), as well as strains 5 (128 mg/L), 6 (128 mg/L), 7 (128 mg/L), 8 (320 mg/L), 9 (128 mg/L), and 10 (128 mg/L), did not result in a statistically significant inhibition (*p* > 0.05). The effect of the sub-MICs of BBR on the inhibition of motility of selected *C. difficile* strains on soft agar is presented in Fig. 3.

**Table 2:** Inhibition of motility of *C. difficile* strains under the influence of different concentrations of berberine

Strain no.		Control (mean mm)		Berberine (mean mm)	
		24 h	48 h	24 h	48 h
1	630	2.3	3	0 (D)	0 (D)
2	ATCC 9689	2	2.7	1.7 (A)	2.7 (A)
3	M 120	3.3	3.7	2 (A)	2 (A)
4*	1468	4.7	10.3	2.7 (B)	3.7 (B)**
5*	3972	5	14	5 (A)	13.3 (A)
6*	898	4	12	3.3 (A)	10.7 (A)
7*	1201	2.6	5.7	2 (A)	8.7 (A)
8*	5473	0	5	0 (B)	4.3 (B)
9*	325	4	9.3	3 (A)	9.3 (A)
10*	443	4.7	11	5 (A)	11 (A)
11*	2292	4.3	10	3 (C)**	4.3 (C)**
12*	5323	5	6.7	2.7 (C)**	4.3 (C)**

*A* BBR 128 mg/L, *B* BBR 320 mg/L, *C* BBR 450 mg/L, *D* 512 mg/L

*Clinical *C. difficile* strains

**Statistically significant

## Discussion

*C. difficile* is the leading multidrug-resistant pathogen in hospital-acquired diarrhea [1]. Currently, CDI is managed via the use of the two conventional antimicrobials, metronidazole and vancomycin, for the treatment of mild to moderate CDI and severe CDI, respectively. Fidaxomicin is a good alternative, especially in patients at risk of relapse [8]. However, several issues are associated with the use of these agents, including a high recurrence rate of 20–25%. *C. difficile* has developed resistance to several different classes of antimicrobials; however, the rate of resistance varies widely depending on the geographic regions and policies of antimicrobial use [24]. Natural products derived from food and other plant extracts have great antimicrobial potential against multi-drug-resistant microorganisms [25]. Berberine is a natural isoquinoline alkaloid that is increasingly drawing attention due to its multiple therapeutic effects, among others, on cancer, diabetes, hyperlipidemia, and cardiovascular diseases [26–29]. In traditional Chinese medicine, berberine has been widely used to treat bacterial diarrhea and gastroenteritis [30]. It has been previously indicated that, within the same species of bacteria, some strains may present distinct sensitivities to antimicrobial agents. Our research revealed that the MIC of berberine against *C. difficile* strains ranged from 256 to 1.024 mg/L. In contrast, Tan et al. showed that the MIC values of berberine varied from 64 to 512 mg/L against *Staphylococcus aureus* [31]. Wang et al. found that the MIC value of berberine for *C. difficile* spores was 640 mg/L [17]. In this study, a higher MBC was observed, possibly due to the fact that some *C. difficile* cells are found in the form of spores, which can wait later. It is likely that berberine will not enter the core of dormant spores due to the core’s extreme impermeability, consistent with the dormant spores’ resistance to antibiotics. The level of berberine accumulated at the berberine MICs in the individual germinated spores was heterogeneous for *C. difficile*. These values were 25–50-fold higher than the MIC values. However, berberine did not affect the germination of *C. difficile* spores, but did block the outgrowth of germinated spores. In our study, a higher MBC (in strain 1) was observed, at 16.384 mg/L. A certain amount of bacteria in special spores of *C. difficile* was able to survive and persist at high berberine concentrations. Our study tested the sensitivity of 9 clinical strains (RT027) to berberine. As a result, we found that berberine presented significant antibacterial activity against all strains. Furthermore, synergism was observed in berberine at 1/2 MICs combined with vancomycin. The great diversity of MICs among the *C. difficile* strains indicates the importance of determining the MIC value. Zuo et al. found that berberine significantly lowered the MIC values of a series of antibiotics against *S. aureus* [32].

In a previous study, the antimicrobial effect of berberine chloride in combination with various anti-staphylococcal drugs on reference CoNS strains was found to vary greatly depending on the bacterial strain and drug used. The most significant synergistic effects towards CoNS strains were noted when berberine was combined with linezolid, cefoxitin, and erythromycin [33]. In another study, the combined use of fusidic acid (FA) and berberine chloride (BBR) was found to result in an in vitro synergistic action against 7 out of 30 clinical methicillin-resistant *Staphylococcus aureus* (MRSA) strains [34]. In the present study, we examined the sub-MICs of berberine and sub-MICs of vancomycin for biofilm formation in *C. difficile* strains. The sub-MICs of BBR and VAN separately did not significantly increase biofilm formation in most strains except one. However, the sub-inhibitory concentration (1/2 MIC) of BBR with VAN was unexpectedly found to enhance biofilm formation in one clinical *C. difficile* strain. This is a form of adaptation by *C. difficile* to highly stressful environments produced by berberine with vancomycin, wherein the organism tended to live in its biofilm form instead of its planktonic form.

The flagella allow bacteria to move and contribute to bacterial colonization and pathogenesis by promoting adhesion to host cells, providing motor-driven nutrients and promoting biofilm formation [35]. The clinical strains tested in this study showed significant motility. Here, three clinical strains were found to have an inhibition of motility after the addition of sub-MIC (1/2 MIC) doses of BBR. The mechanism of action of berberine on the motility of *C. difficile* strains under the influence of sub-inhibitory doses of BBR is interesting: sub-inhibitory doses of BBR reduced the motility of clinical strains to varying degrees. To the best of our knowledge, this is the first study to focus on the biological effect of berberine both alone and in combination with vancomycin on *C. difficile* and biofilm formation.

In conclusion, specific biological substances exhibit antimicrobial properties. Our work demonstrates the antimicrobial ability of berberine against *C. difficile*. Importantly, we observed that sub-MICs of BBR with VAN can enhanced biofilm formation in some *C. difficile* strains.
